# A new, unusually dark, typhlocybine leafhopper (Hemiptera, Cicadellidae, Typhlocybinae, Erythroneurini) from China

**DOI:** 10.3897/zookeys.1042.63593

**Published:** 2021-06-04

**Authors:** Yuehua Song, Zhouwei Yuan, Jia Jiang

**Affiliations:** 1 School of Karst Science, Guizhou Normal University/ State Engineering Technology Institute for Karst Desertification Control, Guiyang, Guizhou 550001, China Guizhou Normal University Guiyang China

**Keywords:** Auchenorrhyncha, Homoptera, morphology, new taxa, taxonomy

## Abstract

An unusually dark typhlocybine leafhopper (Cicadellidae, Typhlocybinae, Erythroneurini) from Guizhou Province, China, is described as a new genus and species, *Shibinga***gen. nov.**, and *S. nigra***sp. nov.** Detailed morphological descriptions and illustrations of the new species are provided.

## Introduction

ErythroneuriniYoung (1952) is the largest tribe in the subfamily Typhlocybinae. The tribe is distinguished by the hind wing without apical submarginal vein (Fig. 2N) and is particularly diverse in the Original region where many genera and species remain to be described. In the course of studying Chinese Erythroneurini, an unusually dark (almost black) colored species was collected from Guizhou Province (Southwest China) and found to belong to a new genus and species, described herein. Most other Typhlocybinae are yellow or green, often with conspicuous markings. In addition to its unusual color, the new species is noteworthy for its short and robust subgenital plate (Fig. 2C) and greatly enlarged style preapical lobe (Fig. 2G, H).

## Materials and methods

Morphological terminology used in this work follows Dietrich (2005). Habitus photos were taken using a KEYENCE VHX-5000 digital microscope. Body length was measured from the apex of the vertex to the tip of the forewings. Abdomens were removed from specimens and cleared in cold 10% KOH solution overnight. The cleared material was rinsed with water and stored in glycerine. An Olympus SZX16 dissecting microscope was used for specimen study and Olympus BX53 stereoscopic microscopes were used for drawing of the dissected male genitalia and wings. All specimens examined are deposited in the collection of the School of Karst Science, Guizhou Normal University, China (**GZNU**).

## Results

### Hemiptera Linnaeus, 1758

**Cicadellidae Latreille, 1825**

**Typhlocybinae Kirschbaum, 1868**

#### Erythroneurini Young, 1952

##### Shibinga gen. nov.

Taxon classificationAnimaliaHemipteraCicadellidae

7C4E5FBA-26F9-5D95-9899-935BB9F82218

http://zoobank.org/C30CC595-A843-4509-A7F4-A50F690C2E5C

###### Type species.

*Shibinga nigra* sp. nov.

###### Description.

Head, pronotum and mesonotum mainly blackish brown. Face brown marked with yellow. Forewings brownish hyaline. Legs yellow. Abdomen dark brown with margins of segments yellow.

Head narrower than pronotum, short; vertex with coronal suture long and distinct; face with frontoclypeus relatively slender, anteclypeus broad, nearly pentagonal. Pronotum broad, with pyramidal anterior margin; posterior margin slightly concave. Forewing with claval vein distinct; outer apical cell much more than twice as long as wide. Hind wing with RA vein present.

Male abdominal apodemes small, not exceeding 3^rd^ sternite.

Male genitalia with pygofer lobe with posterior margin indented apically; with few fine setae and several microtrichia scattered on dorsal and ventral parts in caudal half; with elongate articulated dorsal appendage, extended to near pygofer apex. Subgenital plate short and robust, with few macrosetae laterally in apical half and numerous short stout setae along upper margin and on distal disc in lateral view; several microtrichia on outer surface medially. Style with foot-like apex, with preapical lobe greatly enlarged. Aedeagus relatively small and simple, preatrium and dorsal apodeme well developed, the latter with dorso-lateral corners greatly extended; gonopore subapical on ventral surface. Connective Y-shaped, with stem similar in length to arms, upturned apically; central lobe small.

###### Etymology.

The genus is named after the locality of the type species, Shibing. The gender is feminine.

###### Distribution.

China (Guizhou).

###### Remarks.

The new genus belongs to Dworakowska’s (2002) “*Salka* group” of genera in being almost entirely black in color with the head narrower than the pronotum. In particular it is near *Yakuza* Dworakowska, 2002, based on its short head, male pygofer with articulated dorsal appendage and without enlarged setae; subgenital plate with few macrosetae arranged obliquely; style apex footlike (with 2 points); connective with median anterior lobe and aedeagus short with dorsal apodeme enlarged and shaft lacking processes. However, it can be distinguished in the male genitalia by its short and robust subgenital plate and greatly enlarged style preapical lobe. In addition, the new genus is also similar to *Chujophila* Dworakowska, 1997 in its enlarged preapical lobe of the style and the shape of the aedeagus, but differs in having the connective central lobe present and lateral arms stronger; pygofer ventral appendages absent and dorsal appendages movably articulated basally, not fused to dorsal margin.

##### Shibinga nigra sp. nov.

Taxon classificationAnimaliaHemipteraCicadellidae

528377D1-5513-5D6D-9EEB-EBB9B4AB5308

http://zoobank.org/45DD05A4-BE4C-4F0B-9BAC-399320323F88

Figs 1 , 2

###### Specimens examined.

***Holotype*:** ♂, China, Guizhou Prov., Shibing, Heichong, 24–28.v.2019, coll. Zhouwei Yuan & Chao Tan. ***Paratypes***: China, Guizhou Prov., Shibing: 1♂, same data as holotype; 1♂, Heichong, 22–24.x.2019, coll. Zhouwei Yuan & Xiao Yang; 2♂♂, Niejiayan, 24–28.v.2019, coll. Zhouwei Yuan & Chao Tan; 1♂, Wangjiazhuang, 24–28.v.2019, coll. Zhouwei Yuan & Chao Tan; 3♀♀, Lutianba, 24–28.v.2019, coll. Zhouwei Yuan & Chao Tan.

###### Measurement.

Body length, males 2.6–2.7 mm, females 2.7–2.8 mm.

###### Description.

**Figure 1.**
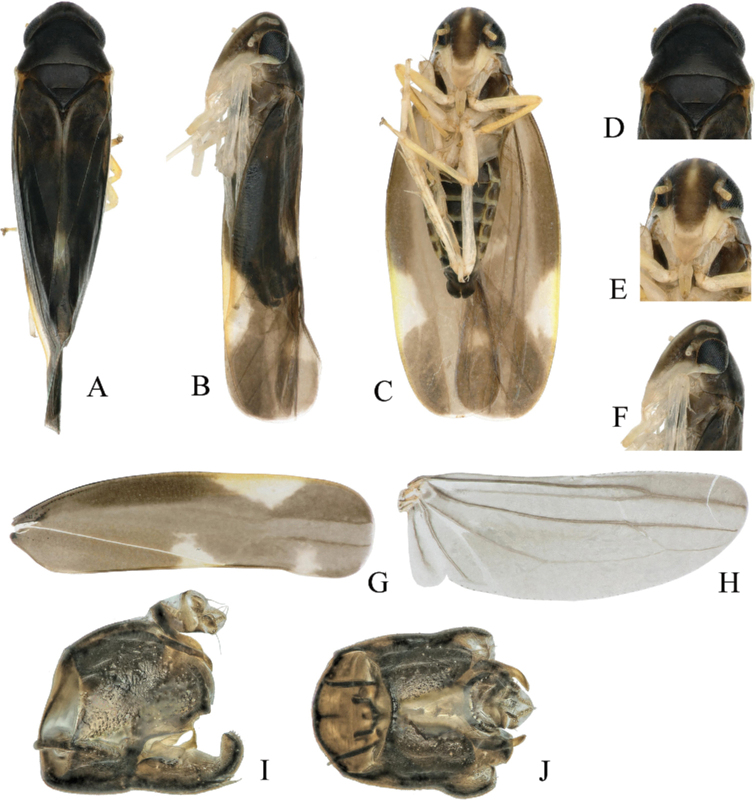
*Shibinga nigra* sp. nov. (♂) **A** habitus, dorsal view **B** habitus, lateral view **C** habitus, ventral view **D** head and thorax, dorsal view **E** face **F** face, lateral view **G** forewing **H** hind wing **I** genital capsule, lateral view **J** genital capsule, dorsal view.

Head, pronotum and mesonotum mainly blackish brown (Fig. 1A–F). Vertex with an irregular yellowish spot on each side at apex, extending onto face. Face dorsally brown, frontoclypeus with a longitudinal medial line in upper half, yellow; anteclypeus brownish yellow; genae yellow (Fig. 1C, E). Forewing dark brownish hyaline with three irregular yellowish white markings as in Fig. 1G. Legs yellow. Abdomen dark brown with margins of segments yellow.

**Figure 2.**
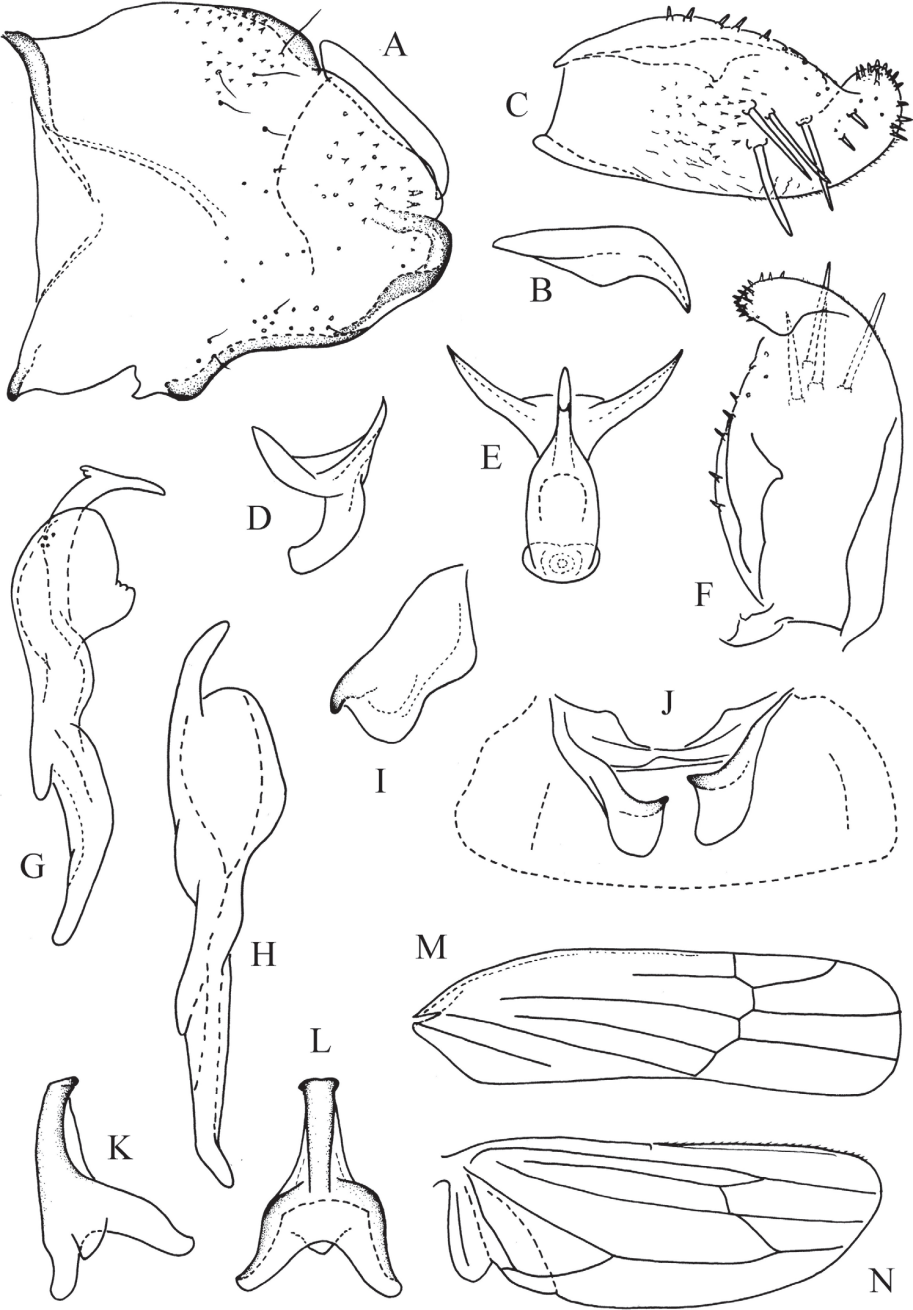
*Shibinga nigra* sp. nov. **A** male pygofer, lateral view **B** pygofer dorsal appendage **C** subgenital plate, ventrolateral view **D** aedeagus, lateral view **E** aedeagus, ventral view **F** subgenital plate, dorso-lateral view **G** style, ventral view **H** style, lateral view **I** left abdominal apodeme, lateral view **J** abdominal apodemes, ventral view **K** connective, dorso-lateral view **L** connective, ventral view **M** forewing **N** hind wing.

Male abdominal sternal apodemes short, not extending to hind margin of 3^rd^ segment (Fig. 2J); broad and expanded in lateral view (Fig. 2I).

Male genitalia as in generic description. Pygofer not extended to apex of subgenital plate, articulated dorsal appendage slightly curved distally (Fig. 2A). Subgenital plate with 4 macrosetae laterally in distal half (Fig. 2C, F). Style preapical lobe greatly enlarged (Fig. 2G, H). Aedeagal shaft short and slim (Fig. 2D); dorsal apodeme with dorsal lateral corners greatly extended and sharp apically (Fig. 2E); preatrium moderately long, little longer or equal to length of shaft (Fig. 2D, E); gonopore subapical on ventral surface (Fig. 2E). Connective Y-shaped (Fig. 2L).

###### Etymology.

The species is named for its unusual dark color, from the Latin niger, black.

###### Remarks.

This species can be distinguished by external and male genitalia characters (see generic remarks).

## Supplementary Material

XML Treatment for Shibinga

XML Treatment for Shibinga nigra
